# A combined tumor burden–based nomogram for synchronous esophageal and gastric cancers: development and external validation

**DOI:** 10.3389/fonc.2026.1890564

**Published:** 2026-09-04

**Authors:** Meng Xie, Jing Wang, Ziyu Jia, Ke Ji, Qi Wu

**Affiliations:** 1 Key Laboratory of Carcinogenesis and Translational Research (Ministry of Education), Endoscopy Center, Peking University Cancer Hospital & Institute, Beijing, China; 2 Key Laboratory of Carcinogenesis and Translational Research (Ministry of Education/Beijing), Gastrointestinal Cancer Center, Peking University Cancer Hospital & Institute, Beijing, China; 3 State Key Laboratory of Holistic Integrative Management of Gastrointestinal Cancers, Endoscopy Center, Peking University Cancer Hospital & Institute, Beijing, China

**Keywords:** esophageal cancer, external validation, gastric cancer, prognostic nomogram, synchronous multiple primary cancers

## Abstract

**Background:**

Synchronous dual-primary esophageal and gastric cancers (SEGC) are rare upper gastrointestinal malignancies presenting substantial challenges for prognostic assessment and individualized clinical evaluation. Conventional single-organ staging systems inadequately capture the cumulative tumor burden of synchronous cancers, limiting accurate prognostic assessment when tumors with different extents coexist. This study aimed to develop and externally validate a prognostic nomogram incorporating a Combined EG_Stage framework for exploratory risk stratification and post-treatment prognostic counseling in actively treated SEGC patients.

**Methods:**

A retrospective development cohort was assembled from a high-volume tertiary cancer center over 10 years (n = 46), with overall survival (OS) as the primary endpoint. A simplified Combined EG_Stage framework was developed to characterize cumulative anatomical tumor extent. Candidate prognostic predictors included age, treatment modality, and Combined EG_Stage. A multivariable Cox proportional hazards model was used to construct the nomogram. External validation used an independent cohort identified from 6,896 patients with multiple primary malignancies in the Surveillance, Epidemiology, and End Results (SEER) database, yielding 88 eligible patients. Model performance was assessed by discrimination, calibration, risk stratification, and decision curve analysis.

**Results:**

The final nomogram incorporated age, treatment modality, and Combined EG_Stage. Internal validation demonstrated satisfactory calibration across 1-, 3-, and 5-year horizons. In the external cohort, the model achieved a C-index of 0.681, with time-dependent AUC values of 0.616, 0.674, and 0.640 for 1-, 3-, and 5-year OS. Risk stratification significantly differentiated survival outcomes (median OS: 41 vs. 22 months; *P* = 0.022). Decision curve analysis suggested limited and exploratory net benefit across selected threshold probabilities, which should be interpreted cautiously given the small sample size.

**Conclusions:**

This study developed and externally evaluated a clinically interpretable but modestly discriminative prognostic nomogram for actively treated SEGC patients. By integrating age, treatment modality as a real-world prognostic indicator, and combined anatomical tumor burden, the model may support exploratory risk stratification in this rare malignancy. Given the limited sample size and only modest discriminative performance, the model should be considered hypothesis-generating rather than definitive, and further multicenter validation is required to assess its robustness and generalizability.

## 1 Introduction

Esophageal and gastric cancers are among the leading causes of cancer-related mortality worldwide, and their synchronous occurrence represents a complex clinical scenario requiring comprehensive prognostic assessment and multidisciplinary management (1). Although synchronous esophageal and gastric cancers (SEGC) remain uncommon, their detection has increased with broader implementation of upper gastrointestinal screening and advances in endoscopic diagnosis (2–5). The coexistence of two primary malignancies in anatomically adjacent yet biologically distinct organs create substantial heterogeneity in tumor burden, disease extent, and clinical outcomes, making individualized prognostic assessment particularly challenging (6, 7).

Current prognostic assessment and clinical characterization of SEGC rely largely on conventional single-organ staging systems, including the American Joint Committee on Cancer (AJCC) and Union for International Cancer Control (UICC) TNM classifications (8, 9). However, these systems were developed for isolated primary tumors and may not adequately account for the cumulative burden of synchronous malignancies. In clinical practice, prognostic interpretation is often influenced by the more advanced lesion, despite substantial heterogeneity in stage combinations between esophageal and gastric tumors. This limitation complicates individualized prognostic assessment and risk stratification, highlighting the need for a framework that better captures the cumulative tumor burden and supports post-treatment outcome-oriented clinical counseling in patients with SEGC who have already received active treatment (9–11).

Existing evidence on SEGC is largely limited to retrospective case series and single-center institutional studies, underscoring the substantial clinical heterogeneity and prognostic uncertainty of this rare disease. The rarity of SEGC further limits the feasibility of assembling adequately characterized cohorts, even in high-volume cancer centers, thereby hindering the development and external validation of clinically interpretable prognostic tools. Although selected patients may undergo multimodal management approaches, including surgery or endoscopic treatment, the absence of a dedicated prognostic framework limits individualized risk assessment and survival estimation in this heterogeneous population (12–14). To date, no widely validated prognostic model has been specifically developed to account for the combined burden of synchronous esophageal and gastric malignancies. Given the lack of dedicated prognostic tools for SEGC, we sought to develop an exploratory clinically interpretable risk framework capable of accounting for the combined burden of synchronous esophageal and gastric tumors. We therefore proposed a Combined EG_Stage system based on the relative extent of tumor invasion across both primary sites and incorporated it into a prognostic nomogram for individualized survival estimation. To assess its generalizability, the model was externally evaluated in an independent population-based cohort derived from the Surveillance, Epidemiology, and End Results (SEER) program.

## 2 Methods

### 2.1 Study design and patient cohorts

This study employed a dual-cohort design consisting of a local development cohort and an independent external validation cohort. For model development, we retrospectively identified consecutive patients diagnosed with synchronous esophageal and gastric cancers (SEGC) at Peking University Cancer Hospital between 2011 and 2021. SEGC was defined according to the Warren and Gates criteria (15), requiring pathologically distinct primary malignancies diagnosed within a six-month interval (16). Patients were eligible if complete baseline clinicopathological data, definitive treatment information, and survival follow-up were available. A total of 46 eligible patients were identified to constitute the local development cohort, reflecting the rarity of SEGC even within a high-volume tertiary cancer center performing more than 10,000 upper gastrointestinal endoscopic examinations annually.

External validation was conducted using the Surveillance, Epidemiology, and End Results (SEER) Research Plus Data, 17 Registries (November 2025 submission). Following sequential filtering of approximately 6,900 patients with multiple primary malignancies in the SEER registry, 100 potentially eligible SEGC cases were initially identified. Because no patients receiving best supportive care alone were present in the local development cohort, individuals who received only best supportive care (BSC) were excluded from the external validation cohort (n = 12). Active treatment was operationally defined as the receipt of cancer-directed surgery (SEER surgery codes 20–90), chemotherapy, or radiotherapy (Beam or Yes). Patients lacking any of these active therapeutic modalities were categorized as receiving BSC. This restriction was applied to improve cohort comparability and to ensure that external validation was performed within the same actively treated population represented in the development cohort. The final external validation cohort included 88 patients with complete treatment data, defined Combined EG_Stage classifications, and available survival outcomes. The exclusion process is detailed in **Supplementary Figure 1** for the SEER cohort and in the main text for the institutional cohort.

For the SEER external validation cohort, synchronous esophageal and gastric malignancies were identified using primary site codes C15.x (esophagus) and C16.0–C16.6 (stomach). Given the clinical ambiguity of tumors at the esophagogastric junction (EGJ), we adopted an inclusive approach: any primary tumor coded as C16.0 (cardia) or C16.1 (fundus) was considered a gastric primary in this study, provided it met the Warren and Gates criteria for synchronous presentation. This definition was chosen to align with standard SEER registry coding practices and to ensure a sufficiently sized validation cohort, acknowledging that EGJ tumors may exhibit biological behaviors intermediate between esophageal and gastric cancers. Detailed SEER selection criteria and coding definitions are provided in Additional file 2: **Supplementary Table 1**.

### 2.2 EG_stage system and variable definitions

To characterize the cumulative burden of synchronous esophageal and gastric tumors in a clinically interpretable manner, we integrated exploratory survival analyses with established oncologic principles. As shown in **Supplementary Figure 2**, esophageal T stage demonstrated a statistically significant association with overall survival (log-rank *P* = 0.014), supporting its potential relevance as a component of tumor burden stratification. In contrast, gastric T stage subcategories and individual nodal (N) status showed heterogeneous and non-significant survival patterns (all *P* > 0.05), reflecting both limited statistical power in these small subgroups and inherent variability in retrospective nodal assessment.

Given these limitations, detailed T/N subclassification was considered insufficiently robust for stable stratification, and a simplified dichotomization strategy was adopted to reduce subgroup fragmentation and improve the robustness of exploratory prognostic stratification. Within this framework, early-stage disease (e) was defined as T1 tumors confined to the mucosa or submucosa without nodal involvement (T1N0), whereas advanced-stage disease (ad) included either deeper T invasion (T2–T4) or any nodal metastasis in T1 tumors (T1N+).

Importantly, classification of T1N+ disease as advanced was not based solely on cohort-specific statistical significance but on established oncologic principles. In gastrointestinal malignancies, regional lymph node involvement at an early T stage represents a key biological transition toward systemic disease, with prognostic behavior more comparable to locally advanced disease (T2–T4) than to T1N0 tumors. Therefore, nodal positivity was incorporated as a binary indicator of biologically meaningful progression rather than as a fine-grained staging variable. This approach prioritizes biological relevance and clinical interpretability over strict anatomical granularity in a small-sample setting.

To ensure rigorous harmonization between the institutional and SEER cohorts, tumor (T) and nodal (N) categories in the SEER registry were derived using the “Derived AJCC T” and “Derived AJCC N” variables. These variables provide standardized staging information across diagnosis years based on SEER’s cross-era AJCC (6th–8th editions) staging algorithms, using available clinical, pathological, and imaging information rather than uniform pathological re-staging. Cases with insufficient or unknown nodal information were handled according to SEER coding rules and excluded from stage reclassification when EG_Stage assignment was not possible.

Patients were subsequently categorized into four Combined EG_Stage groups based on paired esophageal and gastric tumor status: dual-early (eEeG), esophageal-early/gastric-advanced (eEadG), esophageal-advanced/gastric-early (adEeG), and dual-advanced (adEadG). Treatment modality was classified as a prognostic covariate into three groups: endoscopic resection-based treatment (ER/ER+), surgery-based treatment (Surgery/Surgery+), and chemotherapy/chemoradiotherapy (C(R)T).

### 2.3 Statistical analysis and nomogram construction

Overall survival (OS) was calculated from a uniform index date defined as the date of initiation of the first anti-cancer treatment (surgery, chemotherapy, or radiotherapy) for either the esophageal or gastric primary tumor, whichever occurred first, to the date of death or last follow-up. This uniform index date was adopted to minimize immortal time bias that could arise from anchoring survival to a later diagnostic event, given the allowable 6-month diagnostic interval between the two primaries.

Missing data were handled using a complete-case analysis approach. Because incomplete records occurred primarily at the initial screening stage, patients with missing key variables required for staging, treatment classification, or survival outcomes were excluded prior to analysis; therefore, no missing data were present in the final analytic cohorts. In the local development cohort, OS was estimated using the Kaplan-Meier method, and group differences were assessed using the log-rank test. Univariable Cox proportional hazards regression was used to screen candidate predictors of OS.

Treatment modality was included as a composite prognostic variable reflecting real-world treatment selection, underlying disease severity, and clinical management context. Therefore, associations between treatment modality and survival outcomes were interpreted within a prognostic rather than causal framework and were not considered evidence of treatment efficacy or guidance for treatment selection.

Given the rarity of SEGC and the limited number of outcome events, model complexity was deliberately constrained to develop an exploratory prognostic stratification model while minimizing overfitting risk under a low events-per-variable (EPV) setting. Multivariable model selection was performed using backward stepwise Cox regression guided by the Akaike Information Criterion (AIC), recognizing the potential for model instability in small datasets. To quantify and correct for potential optimism in model performance, internal validation was performed using bootstrap resampling with 1,000 iterations. In each iteration, a bootstrap sample was drawn with replacement from the original dataset, the model was refitted, and its performance was evaluated both in the bootstrap sample and in the original dataset. Optimism was defined as the average difference between bootstrap performance (apparent performance in bootstrap sample) and test performance (performance in original dataset). The optimism-corrected C-index was obtained by subtracting the estimated optimism from the apparent C-index. To assess model robustness and parsimony under EPV constraints, a single pre-specified sensitivity analysis was conducted comparing the final multivariable model with a reduced model including only Combined EG_Stage and Age. Discriminative performance was compared using the apparent C-index. The proportional hazards assumption was evaluated using Schoenfeld residuals, and all variables in the final model satisfied the assumption (P > 0.05). An exploratory prognostic nomogram was constructed based on the final multivariable Cox model to facilitate individualized survival estimation and risk stratification. Model discrimination was assessed using Harrell’s C-index and time-dependent AUC at 1-, 3-, and 5-year time points. Internal calibration was evaluated using bootstrap resampling (1,000 repetitions). External validation was performed in an independent SEER cohort without model refitting to evaluate the generalizability of the exploratory prognostic framework. For external calibration, smoothed calibration curves based on restricted cubic splines were used to avoid instability associated with small sample binning. Mean absolute error (MAE) was calculated to quantify predictive deviation. Patients in the external cohort were stratified into exploratory risk groups based on the median linear predictor derived from the development cohort, and survival differences were evaluated using the log-rank test to assess prognostic separation. Clinical utility was assessed using decision curve analysis. All statistical analyses were performed using R software (version 4.3.0; R Foundation for Statistical Computing, Vienna, Austria) with the survival, rms, timeROC, and polspline packages. All tests were two-sided, and P < 0.05 was considered statistically significant.

## 3 Results

### 3.1 Baseline characteristics

Following exclusion of patients without active cancer-directed treatment, 88 SEER patients were included for external validation. Baseline characteristics of the local development cohort (n = 46) and the SEER validation cohort (n = 88) are summarized in **Table 1**. Median age was comparable between cohorts (66.0 years [IQR, 61.0–70.0] vs. 67.0 years [IQR, 61.5–75.5]), and both cohorts were predominantly male (89.1% vs. 83.0%). Differences were observed in Combined EG_Stage distribution: eEadG disease was most common in the local cohort (41.3%), whereas eEeG disease predominated in the SEER cohort (34.09%). Median follow-up duration was broadly comparable between cohorts (30.1 months [IQR, 10.9–51.4] vs. 32.5 months [IQR, 14.0–82.0]).

**Table 1:** Baseline clinicopathological characteristics of local and SEER cohorts.

Characteristic	Local development cohort (N = 46)	SEER validation cohort (N = 88)
Age (years)	66.00 (61.00 - 70.00)	67.00 (61.50 - 75.50)
Gender		
Male	41 (89.13%)	73 (82.95%)
Female	5 (10.87%)	15 (17.05%)
Combined EG_stage		
adEadG	12 (26.09%)	22 (25.00%)
adEeG	7 (15.22%)	15 (17.05%)
eEadG	19 (41.30%)	21 (23.86%)
eEeG	8 (17.39%)	30 (34.09%)
Follow-up Duration (months)	30.13 (10.93 - 51.43)	32.50 (14.00 - 82.00)
Treatment modality		
ER/ER+	15 (32.61%)	16 (18.18%)
Surgery/Surgery+	17 (36.96%)	45 (51.14%)
C(R)T	14 (30.43%)	27 (30.68%)
Tumor location (Esophagus)		
Cervical esophagus	2 (4.35%)	1 (1.14%)
Thoracic esophagus	13 (28.26%)	13 (14.77%)
Abdominal esophagus	25 (54.35%)	66 (75.00%)
Esophagus, NOS	6 (13.04%)	8 (9.09%)
Tumor location (Stomach)		
Cardia or fundus of stomach	24 (52.17%)	59 (67.05%)
Body of stomach	4 (8.70%)	10 (11.36%)
Gastric antrum or pylorus	16 (34.78%)	7 (7.95%)
Stomach, NOS	2 (4.35%)	12 (13.64%)

Data are presented as median (interquartile range [IQR]) or n (%). Abbreviations: EG_Stage, combined esophagogastric stage; eEeG, early esophageal and early gastric cancer; eEadG, early esophageal and advanced gastric cancer; adEeG, advanced esophageal and early gastric cancer; adEadG, advanced esophageal and advanced gastric cancer; ER/ER+, endoscopic resection alone or combined with surgery; Surgery/Surgery+, surgery with or without adjuvant treatment; C(R)T, chemotherapy and/or radiotherapy.

### 3.2 Prognostic factors associated with overall survival

**Figure 1:**
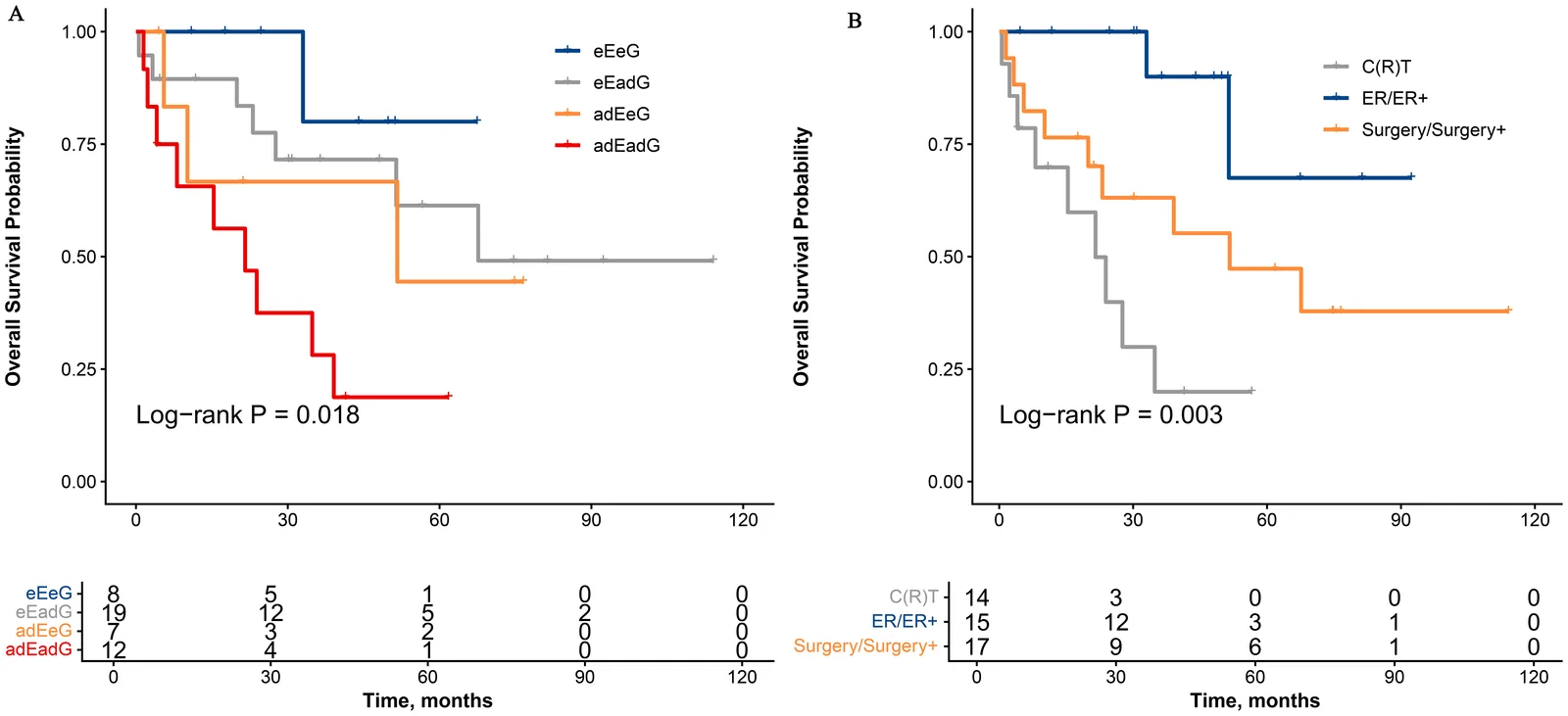
Kaplan–Meier overall survival curves stratified by **(A)** Combined EG_Stage and **(B)** treatment modality in the local development cohort. Patients with dual early-stage disease (eEeG) showed the longest observed survival, whereas those with dual advanced-stage disease (adEadG) showed the shortest observed survival. Survival differences across treatment modality groups are presented descriptively, as treatment allocation reflects heterogeneous clinical scenarios, underlying disease characteristics, and treatment selection rather than comparable therapeutic strategies. Tick marks indicate censored observations.

Kaplan–Meier analyses demonstrated significant differences in overall survival according to both Combined EG_Stage (log-rank *P* = 0.018; **Figure 1A**) and across treatment modality categories (*P* = 0.0033; **Figure 1B**). Patients with dual early-stage disease (eEeG) exhibited the most favorable observed survival pattern, whereas dual advanced-stage disease (adEadG) showed the least favorable survival pattern.

Candidate variables for multivariable modeling were selected based on clinical relevance, interpretability, availability across cohorts, and exploratory associations with overall survival (**Table 2**). Given the limited number of death events in the development cohort (n = 20), the number of candidate predictors was intentionally restricted to reduce overfitting and preserve model stability. Only clinically meaningful macroscopic variables with adequate availability across both cohorts—including age, Combined EG_Stage, treatment modality, and pretreatment Karnofsky Performance Status (KPS)—were initially considered.

**Table 2:** Univariable and multivariable Cox proportional hazards regression analyses for overall survival.

Variables	Univariate HR (95% CI)	*P* value	Multivariate HR (95% CI)	*P* value
Age (per year)	1.07 (1.00–1.14)	0.04	1.04 (0.97–1.12)	0.22
Treatment modality				
C(R)T	1.00 (Reference)	—	1.00 (Reference)	—
ER/ER+	0.10 (0.02–0.49)	0.00	0.23 (0.03–1.59)	0.14
Surgery/Surgery+	0.44 (0.17–1.16)	0.10	0.66 (0.21–2.12)	0.49
Combined EG_stage				
eEeG	1.00 (Reference)	—	1.00 (Reference)	—
eEadG	2.62 (0.32–21.57)	0.37	1.60 (0.16–15.53)	0.69
adEeG	3.30 (0.34–32.50)	0.31	1.50 (0.11–20.29)	0.76
adEadG	8.88 (1.12–70.22)	0.04	3.00 (0.28–32.75)	0.37
Pretreatment KPS	0.89 (0.81–0.98)	0.02	—	—
Esophageal tumor location				
Thoracic	1.00 (Reference)	—	—	—
Cervical	0.50 (0.06–4.07)	0.52		
Abdominal	0.31 (0.11–0.86)	0.02		
NOS	4.05 (1.13–14.54)	0.03		
Gastric tumor location				
Antrum/Pylorus	1.00 (Reference)	—	—	—
Body	1.04 (0.21–5.05)	0.96	—	—
Cardia/Fundus	0.93 (0.35–2.45)	0.88	—	—
NOS	0.94 (0.12–7.71)	0.96	—	—

HR, hazard ratio; CI, confidence interval; Ref, reference; KPS, Karnofsky Performance Status; NOS, not otherwise specified; EG_Stage, combined esophagogastric stage; eEeG, early esophageal and early gastric cancer; eEadG, early esophageal and advanced gastric cancer; adEeG, advanced esophageal and early gastric cancer; adEadG, advanced esophageal and advanced gastric cancer; ER/ER+, endoscopic resection alone or combined with surgery; Surgery/Surgery+, surgery with or without adjuvant therapy; C(R)T, definitive chemotherapy and/or radiotherapy.

Final model specification was determined using backward stepwise selection guided by the Akaike Information Criterion (AIC), with emphasis on maintaining model parsimony and external applicability. Pretreatment KPS was not retained after optimization. Although KPS demonstrated prognostic association in univariable analysis, its exclusion also enhanced methodological consistency across cohorts because performance-status measures are frequently unavailable or inconsistently recorded in population-based registries such as SEER.

The final multivariable Cox model included age, Combined EG_Stage, and treatment modality. Variable retention prioritized overall predictive contribution and clinical plausibility rather than statistical significance. Treatment modality should be interpreted as a surrogate marker of clinical status and treatment selection rather than a causal determinant of survival. Proportional hazards assumptions were evaluated using Schoenfeld residuals, and no violations were detected for age, Combined EG_Stage, or treatment modality (all *P* > 0.05).

Survival patterns based on conventional single-organ TNM components are shown in **Supplementary Figure 2**. While certain parameters, particularly esophageal T stage, demonstrated exploratory prognostic separation, several highly granular staging variables showed inconsistent or limited discrimination within this dual-primary cohort. Given the heterogeneity and limited event number, these findings supported the development of a simplified Combined EG_Stage framework to capture overall tumor burden in subsequent multivariable modeling.

**Figure 2:**
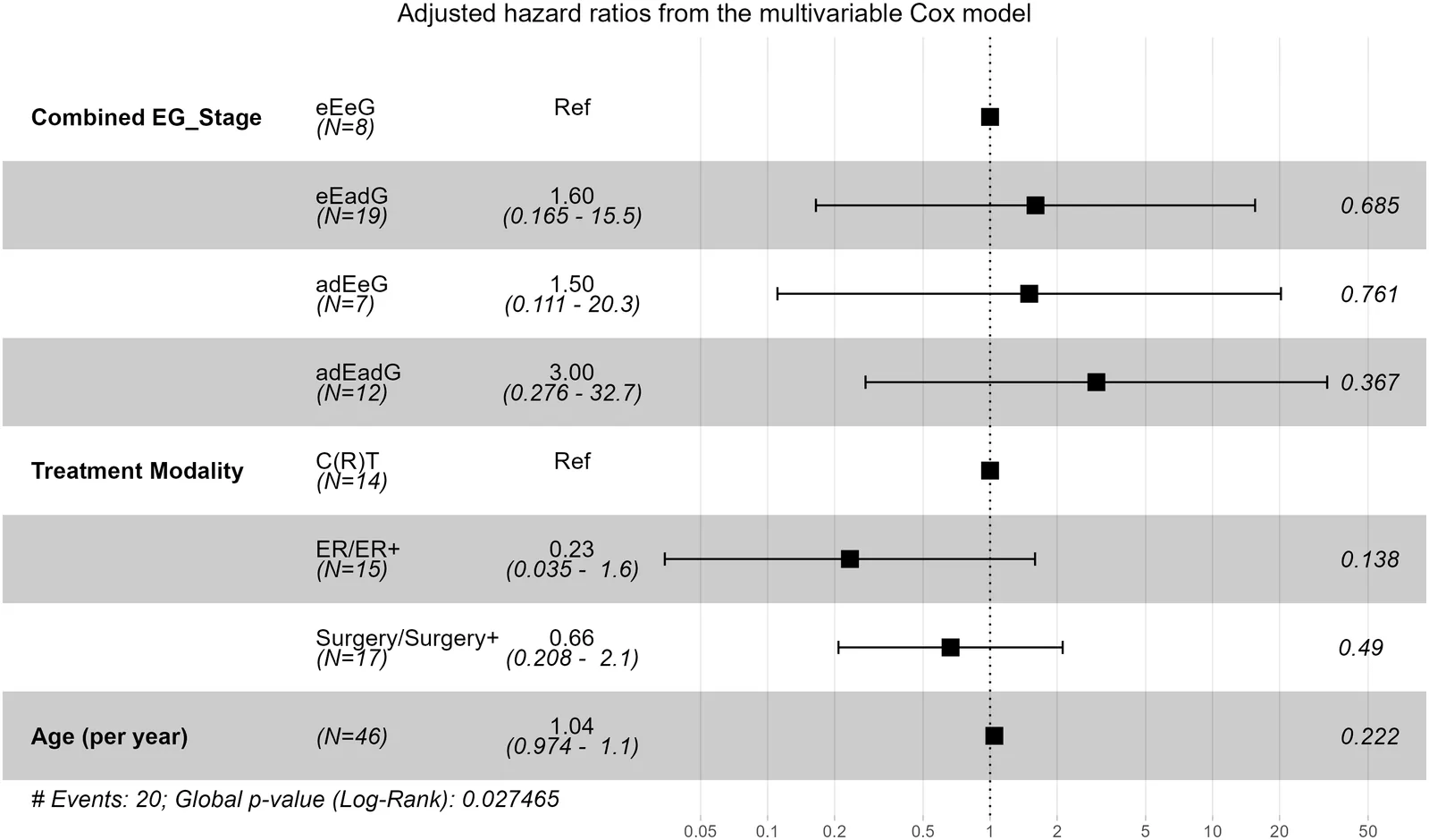
Forest plot of the final multivariable Cox proportional hazards model for overall survival. The model incorporated age, combined EG_stage, and treatment modality as prognostic covariates. Squares indicate adjusted hazard ratios (HRs), and horizontal bars represent 95% confidence intervals (CIs). The vertical dashed line indicates an HR of 1.0. Reference categories were dual early-stage disease (eEeG) and C(R)T. Treatment modality was included as a composite prognostic variable reflecting real-world treatment selection and underlying disease characteristics, and its hazard ratios should not be interpreted as causal estimates of treatment efficacy.

Given the rarity of SEGC and the limited number of outcome events (n = 20), confidence intervals in the multivariable model remained relatively wide, and individual covariates did not achieve conventional statistical significance. Nevertheless, age, Combined EG_Stage, and treatment modality were retained for nomogram construction because the objective was exploratory prognostic stratification rather than formal etiologic inference. The corresponding multivariable hazard ratios are summarized in **Figure 2**.

### 3.3 Nomogram development and internal performance

**Figure 3:**
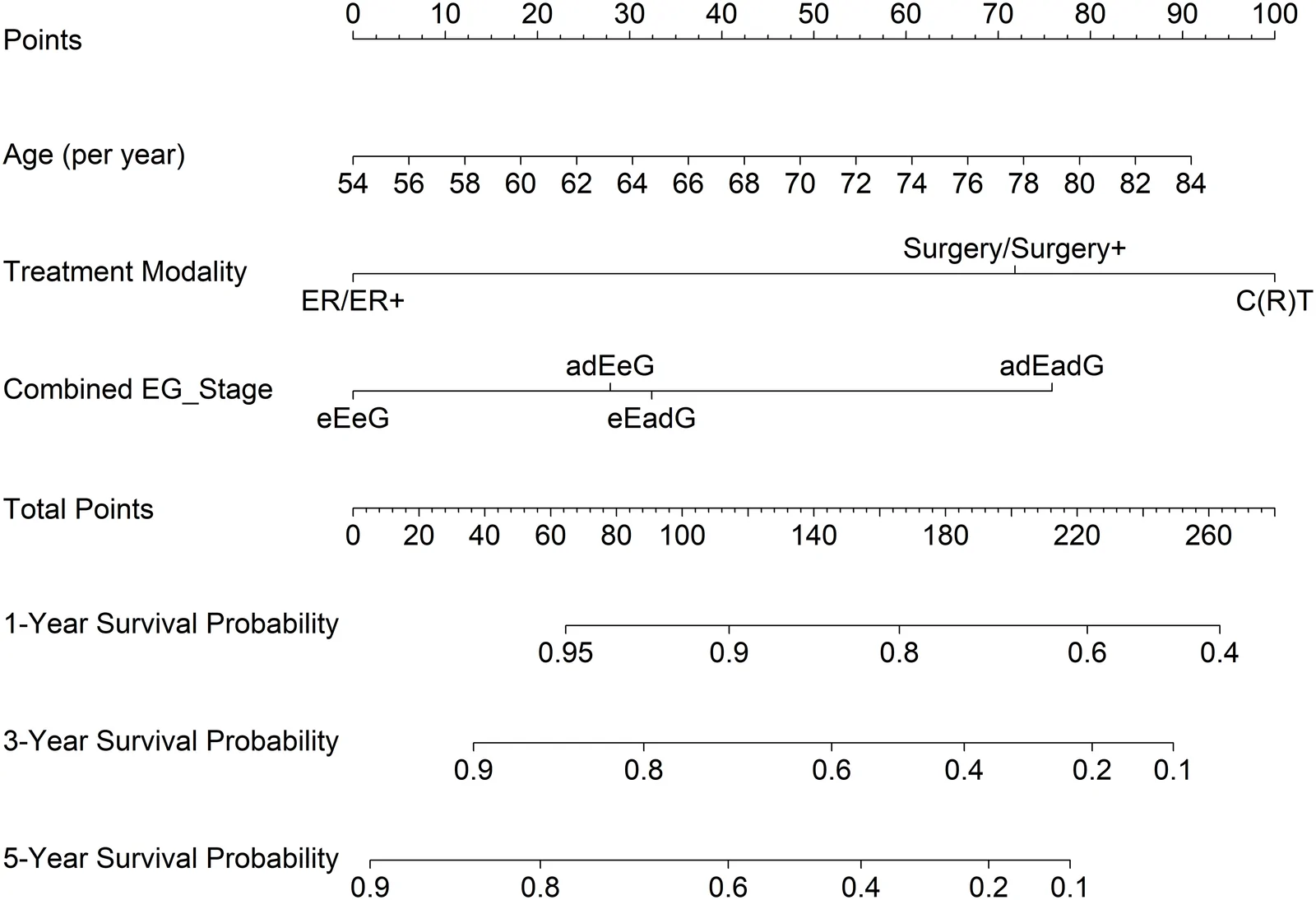
Prognostic nomogram for estimating 1-, 3-, and 5-year overall survival in actively treated patients with synchronous esophageal and gastric cancers. The prognostic nomogram incorporates age, combined EG_Stage, and treatment modality as predictors selected from the final multivariable Cox model. Individual scores are assigned for each predictor, summed to generate a total prognostic score, and used to estimate model-derived survival probabilities.

Based on the final multivariable Cox model, a prognostic nomogram integrating Age, Combined EG_Stage, and Treatment Modality (as a composite indicator of clinical status and treatment selection) was developed to facilitate exploratory prognostic stratification and support post-treatment prognostic counseling among actively treated patients with SEGC (**Figure 3**). Within the nomogram, Combined EG_Stage contributed substantially to the overall point allocation, reflecting its role in capturing the joint burden of synchronous esophageal and gastric malignancies.

**Figure 4:**
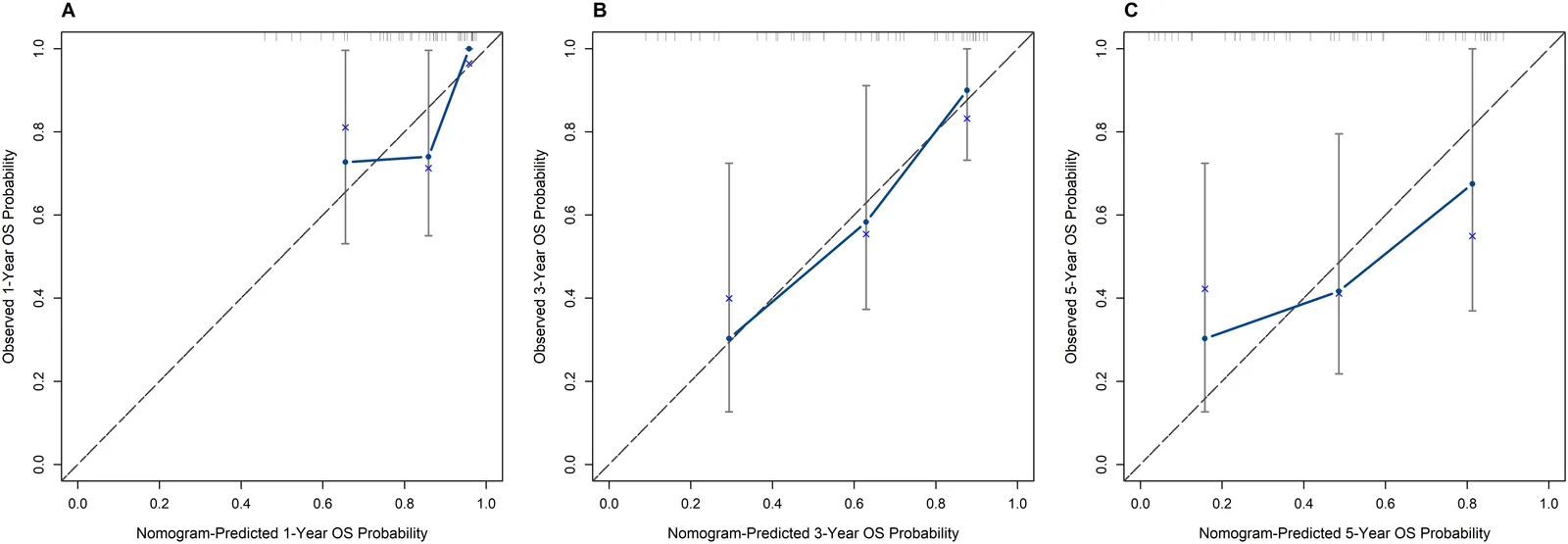
Internal calibration of the prognostic nomogram for predicting overall survival in the local development cohort. Calibration plots for predicted versus observed overall survival at 1 year **(A)**, 3 years **(B)**, and 5 years **(C)** after 1000-bootstrap resampling correction. The diagonal dashed line represents ideal agreement between predicted and observed survival probabilities. Points indicate observed survival estimates across risk groups, and vertical bars represent corresponding uncertainty intervals. Overall, the nomogram showed relatively closer agreement between predicted and observed survival probabilities at the 3-year horizon; however, calibration performance should be interpreted cautiously given the limited sample size and event number.

Internal calibration analyses demonstrated reasonable agreement between predicted and observed overall survival probabilities at 1-, 3-, and 5-year time points (**Figure 4**). Following 1000-bootstrap resampling correction, the nomogram showed the strongest calibration at the 3-year horizon, with acceptable agreement maintained at 1 and 5 years. Mean absolute errors (MAEs) were 7.8%, 2.6%, and 11.8% at 1, 3, and 5 years, respectively. Although modest deviations from the ideal calibration line were observed at shorter and longer follow-up intervals, no substantial systematic miscalibration was identified.

To further assess internal discriminative performance, patients were dichotomized into low-risk and high-risk groups according to the median prognostic score of the local development cohort generated from the finalized nomogram. (**Supplementary Figure 3**). The model demonstrated clear separation of survival trajectories between risk strata, supporting its ability to separate patients into prognostically distinct groups.

As a supplementary exploratory analysis, decision curve analysis (DCA) was performed at the 3-year and 5-year survival horizons (**Supplementary Figures 4A, B**). The Combined EG_Stage model demonstrated modest discrimination and limited exploratory net benefit in decision curve analysis. No clear or consistent superiority over alternative staging systems was observed. Its main contribution is the provision of a simplified and clinically interpretable framework that may assist exploratory risk stratification in this rare disease setting. Given the limited sample size, interpretation of decision curve analysis was restricted to the 10–70% threshold probability range, where sufficient event density was present to support more stable estimation of net benefit. To assess potential overfitting, internal validation using 1000 bootstrap resampling was performed to compare the performance of the primary 3-variable model with a pre-specified parsimonious 2-variable model. The 3-variable model yielded an apparent C-index of 0.753 and an optimism-corrected C-index of 0.642. The 2-variable model demonstrated an apparent C-index of 0.729 and an optimism-corrected C-index of 0.687. Given the limited sample size and number of outcome events, these findings should be interpreted as hypothesis-generating rather than definitive evidence supporting clinical implementation.

### 3.4 External validation in the SEER cohort

**Figure 5:**
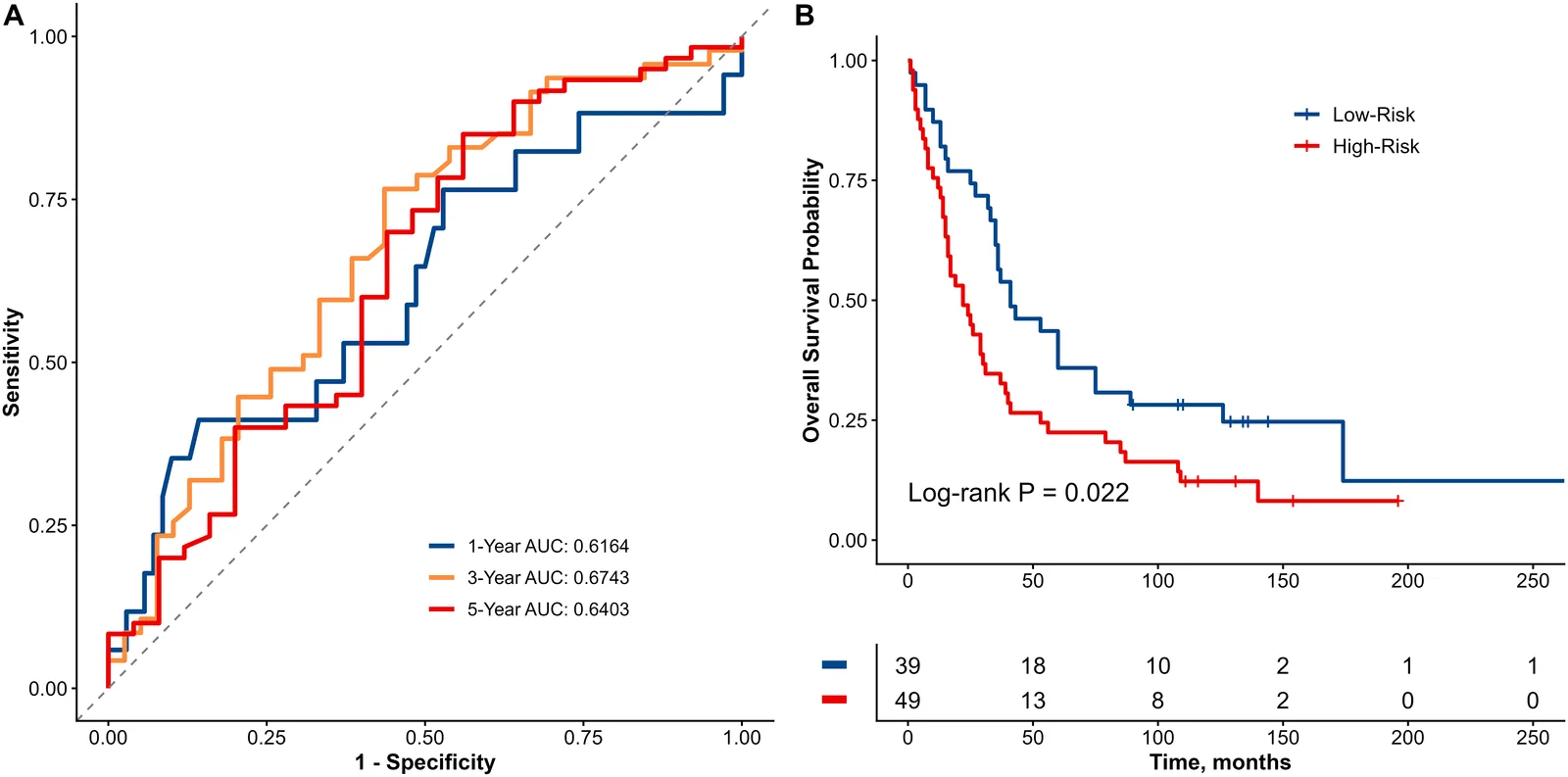
External validation of the prognostic nomogram in the SEER cohort. **(A)** Time-dependent ROC curves illustrating the discrimination performance of the nomogram at 1-, 3-, and 5-year overall survival. Reported AUC values indicate moderate discriminative ability across the evaluated time points. **(B)** Kaplan–Meier curves illustrating exploratory prognostic stratification into low-risk (blue) and high-risk (red) groups using the median nomogram-derived risk score from the local development cohort. Survival distributions differed significantly between the low-risk and high-risk groups (log-rank *P* = 0.022). Tick marks indicate censored observations.

External validation was performed in an independent SEER cohort restricted to actively treated patients (n = 88) without model refitting. The nomogram achieved a concordance index (C-index) of 0.681, with time-dependent AUCs of 0.616, 0.674, and 0.640 for predicting 1-, 3-, and 5-year overall survival estimates, respectively (**Figure 5A**). Importantly, the external C-index (0.681) achieved by the 3-variable primary model aligned remarkably well with the optimism-corrected C-index of the parsimonious model (0.687). These findings suggest the primary model retained comparable discrimination in the external cohort despite the optimism observed during internal validation. However, given the limited sample size and cohort heterogeneity, the external performance should be interpreted as supportive evidence for exploratory prognostic stratification rather than definitive evidence of model generalizability. Using the median nomogram-derived risk score from the local cohort as the predefined cutoff, SEER patients were stratified into low-risk (n = 39) and high-risk (n = 49) groups. Kaplan–Meier analysis demonstrated significantly different survival outcomes between risk strata (log-rank *P* = 0.022), with a median overall survival of 41 months (95% CI, 35–75) in the low-risk group and 22 months (95% CI, 16–37) in the high-risk group (**Figure 5B**).

**Figure 6:**
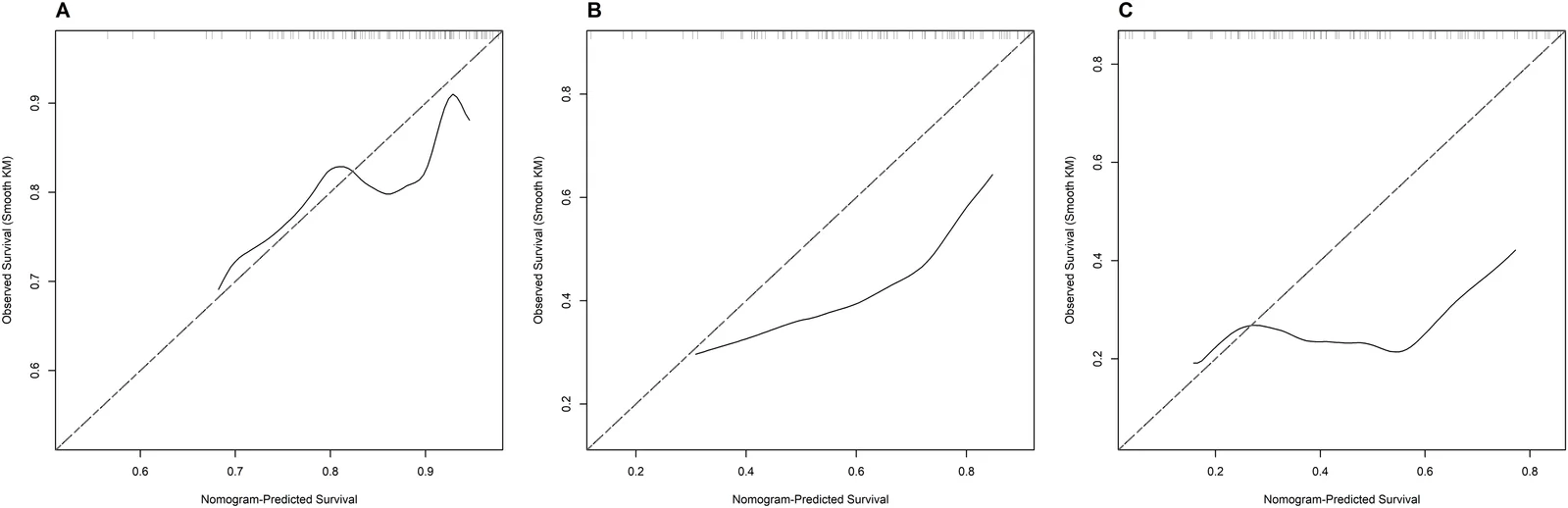
External calibration of the prognostic nomogram in the SEER validation cohort. Calibration plots for 1-year **(A)**, 3-year **(B)**, and 5-year **(C)** overall survival (OS) in the external SEER validation cohort (n = 88). Observed survival probabilities were estimated using smoothed Kaplan–Meier methods, while predicted survival estimates were derived from the final multivariable Cox model. Calibration curves were generated using a restricted cubic spline-based smoothing approach combined with bootstrap resampling (1,000 iterations) to mitigate instability related to small sample size and reduce discretization bias associated with grouped calibration. The dashed diagonal line represents ideal agreement between predicted and observed outcomes. Solid lines indicate smoothed calibration trends for each time horizon. Tick marks on the x-axis represent individual patient-level predicted probabilities. This approach allows continuous assessment of model calibration in a small external cohort, where conventional bin-based calibration may produce unstable or stepwise ("zigzag") patterns because of limited sample density within risk strata.

To address potential grouping artifacts associated with conventional categorical binning in small validation samples, external calibration was assessed using continuous smoothed calibration curves based on restricted cubic splines (the HARE method). These analyses demonstrated acceptable agreement between predicted and observed survival probabilities, with mean absolute errors (MAEs) of 5.41%, 2.35%, and 3.95% at 1, 3, and 5 years, respectively (**Figure 6**). Deriving these metrics directly from continuous smoothed curves rather than categorized risk groups provides a less discretization-dependent assessment of external calibration.

## 4 Discussion

This study presents, to our knowledge, the first externally evaluated prognostic framework specifically developed for exploratory risk stratification in synchronous esophageal and gastric cancers (SEGC). Although the development cohort was modest in size (n = 46), this reflects the inherent rarity of synchronous esophageal and gastric cancers and the difficulty of assembling adequately characterized cohorts for this uncommon disease. The local cohort was assembled from a high-volume tertiary cancer center performing over 10,000 upper gastrointestinal endoscopic examinations annually, with established pathological confirmation procedures and longitudinal follow-up. Likewise, the external validation cohort required extensive filtering from nearly 6,900 patients with multiple primary malignancies within the SEER registry to identify 88 eligible, actively treated SEGC cases. These observations underscore the practical challenge of constructing adequately characterized cohorts for this uncommon dual-primary disease and contextualize the limited event number observed in the present study. Therefore, the proposed nomogram should be interpreted as an exploratory prognostic stratification tool and a hypothesis-generating framework rather than a model intended for treatment selection or clinical decision-making.

Given the rarity and clinical heterogeneity of SEGC, prognostic assessment has traditionally relied on single-organ staging systems, with overall risk often interpreted according to the more advanced lesion (9–11, 17, 18). We therefore proposed a simplified Combined EG_Stage framework to jointly characterize tumor burden across both primary sites and incorporated it into an exploratory prognostic stratification model (19). Hazard ratios associated with treatment modality primarily reflect treatment allocation patterns and underlying disease severity in retrospective datasets, rather than comparative treatment efficacy.

External validation in an independent SEER cohort showed moderate discrimination (C-index = 0.681), together with significant survival separation between predefined risk strata (median OS: 41 vs. 22 months; *P* = 0.022). In internal validation, while our primary 3-variable model (including Combined EG_Stage, Age, and Treatment Modality) yielded an internally optimism-corrected C-index of 0.642, a pre-specified parsimonious 2-variable model (excluding treatment modality) achieved a higher internally optimism-corrected C-index of 0.687. This phenomenon directly reflects the statistical penalty for overfitting induced by introducing an additional variable (Treatment Modality) into a cohort with a low events-per-variable (EPV) ratio. We explicitly acknowledge that from a pure statistical modeling perspective, the 2-variable model demonstrated superior internally penalized discrimination. However, the 3-variable model was retained as the primary prognostic tool because treatment modality provides clinically relevant prognostic information reflecting real-world treatment selection and disease severity in actively treated patients, and may assist in post-treatment discussions of expected prognosis with patients who have already completed active treatment. Notably, despite the internal statistical penalty, the 3-variable model’s external performance (apparent C-index = 0.681) was similar to the optimism-corrected performance of the parsimonious model (0.687). This observation suggests that the primary model retained comparable discriminatory performance in the external cohort, although further validation in larger and more diverse populations is required. Despite differences in healthcare systems and population characteristics, the model retained exploratory prognostic stratification capability in the external cohort, supporting further investigation in larger multicenter datasets.

Calibration performance showed some variation across time horizons, with relatively higher calibration deviation at earlier time points. This may reflect heterogeneity in patient characteristics, disease presentation, and treatment selection patterns between the development and validation cohorts, although further investigation is required to clarify the underlying reasons. Therefore, the observed calibration performance should be interpreted within the context of the specific validation population, and additional external evaluations are required before considering broader application.

Previous studies of SEGC have largely consisted of retrospective case series or institutional experiences, primarily describing surgical feasibility, endoscopic management, or survival outcomes in selected populations (13, 14, 20–23). Although these reports highlight substantial clinical heterogeneity, few have attempted exploratory prognostic modeling, and no externally validated framework integrating tumor burden from both esophageal and gastric primaries has been reported (24–32). Our study extends existing evidence by proposing an interpretable staging-informed nomogram and externally evaluating its performance in an independent population-based cohort.

Several limitations of this study should be acknowledged. First, the development cohort was small and retrospective (n = 46, 20 observed events), reflecting the extreme rarity of synchronous esophageal and gastric cancers and the challenges of assembling adequately characterized cohorts. Based on the number of candidate predictors included in the final multivariable model, the effective events-per-variable (EPV) was approximately 3–4, which is below the commonly cited thresholds for stable regression modeling. Therefore, model estimates should be interpreted as exploratory and potentially subject to overfitting. Although bootstrap resampling was applied to reduce optimism in performance estimation, both discrimination and calibration metrics may still be affected by instability inherent to limited EPV settings. Accordingly, the reported calibration curves and risk stratification results should be considered hypothesis-generating rather than definitive evidence of clinical utility.

Second, the observed discrimination performance in the external SEER cohort was modest (C-index = 0.681 with time-dependent AUCs in the range of 0.61–0.67), indicating limited discriminatory ability that requires further evaluation in larger cohorts. The primary value of the model at this stage lies in exploratory risk stratification and hypothesis generation rather than treatment decision-making or immediate clinical implementation. Further refinement and validation in larger, multicenter cohorts are warranted.

Third, calibration assessment may be influenced by small sample size and risk distribution heterogeneity in the external cohort. Although smoothed calibration methods were used to reduce instability associated with arbitrary risk grouping, uncertainty remains in regions with limited observations because of sparse validation data. Therefore, the external calibration results should be interpreted as supportive evidence of model transportability rather than definitive confirmation of clinical performance.

Fourth, substantial case-mix differences were observed between the development and validation cohorts, with distinct distributions of Combined EG_Stage (eEadG predominance in the local cohort versus eEeG predominance in SEER). This heterogeneity may partially explain differences in calibration across time horizons and suggests that hazard estimates derived from the development cohort may require recalibration before application to populations with different case-mix distributions. Future studies involving larger, multicenter cohorts with broader stage distributions are needed to further evaluate model stability and recalibration strategies.

Fifth, the exclusion of patients receiving best supportive care in the SEER cohort may introduce selection bias, as the validation results reflect actively treated patients rather than an unselected population of all SEGC cases. Finally, the Combined EG_Stage framework, although clinically interpretable, represents a simplified dichotomization of tumor burden and may not fully capture biological heterogeneity. Prospective multicenter studies with larger and more balanced cohorts will be needed to further evaluate model stability, recalibrate performance across diverse populations, and refine staging granularity.

In conclusion, this study proposes an externally evaluated prognostic nomogram for synchronous esophageal and gastric cancers (SEGC) based on a simplified Combined EG_Stage framework. By integrating age, treatment modality, and cumulative tumor burden across both primary sites, the model provides an exploratory framework for estimating survival probabilities using routinely available clinical variables, addressing the current lack of dedicated prognostic models for this rare malignancy. Importantly, treatment-related associations in this model should be interpreted within a prognostic rather than causal framework, as treatment modality reflects heterogeneous clinical indications and underlying tumor burden. Given the limited number of outcome events, the effective events-per-variable ratio was low, which may limit the stability of multivariable regression coefficients. Therefore, estimates of individual predictor effects, particularly Combined EG_Stage categories, should be interpreted cautiously. Although bootstrap-based internal validation was performed, residual optimism cannot be fully excluded. These findings support further evaluation of this exploratory prognostic framework through larger, prospective, multicenter validation studies.

## Data Availability

The SEER dataset analyzed in this study is publicly available from the Surveillance, Epidemiology, and End Results Program. The local institutional dataset generated and analyzed during the current study is not publicly available due to institutional data protection and patient privacy considerations, but de-identified data may be available from the corresponding author upon reasonable request and with appropriate institutional approval.
