# Supplementary material for: A combined tumor burden–based nomogram for synchronous esophageal and gastric cancers: development and external validation
Source: Front Oncol. 2026 Sep 4;16:1890564. doi: 10.3389/fonc.2026.1890564 (PMC13585519; doi:10.3389/fonc.2026.1890564)
Supplement: Supplementary file 1 [file DataSheet1.pdf]

Supplementary Material

1 Supplementary Figures

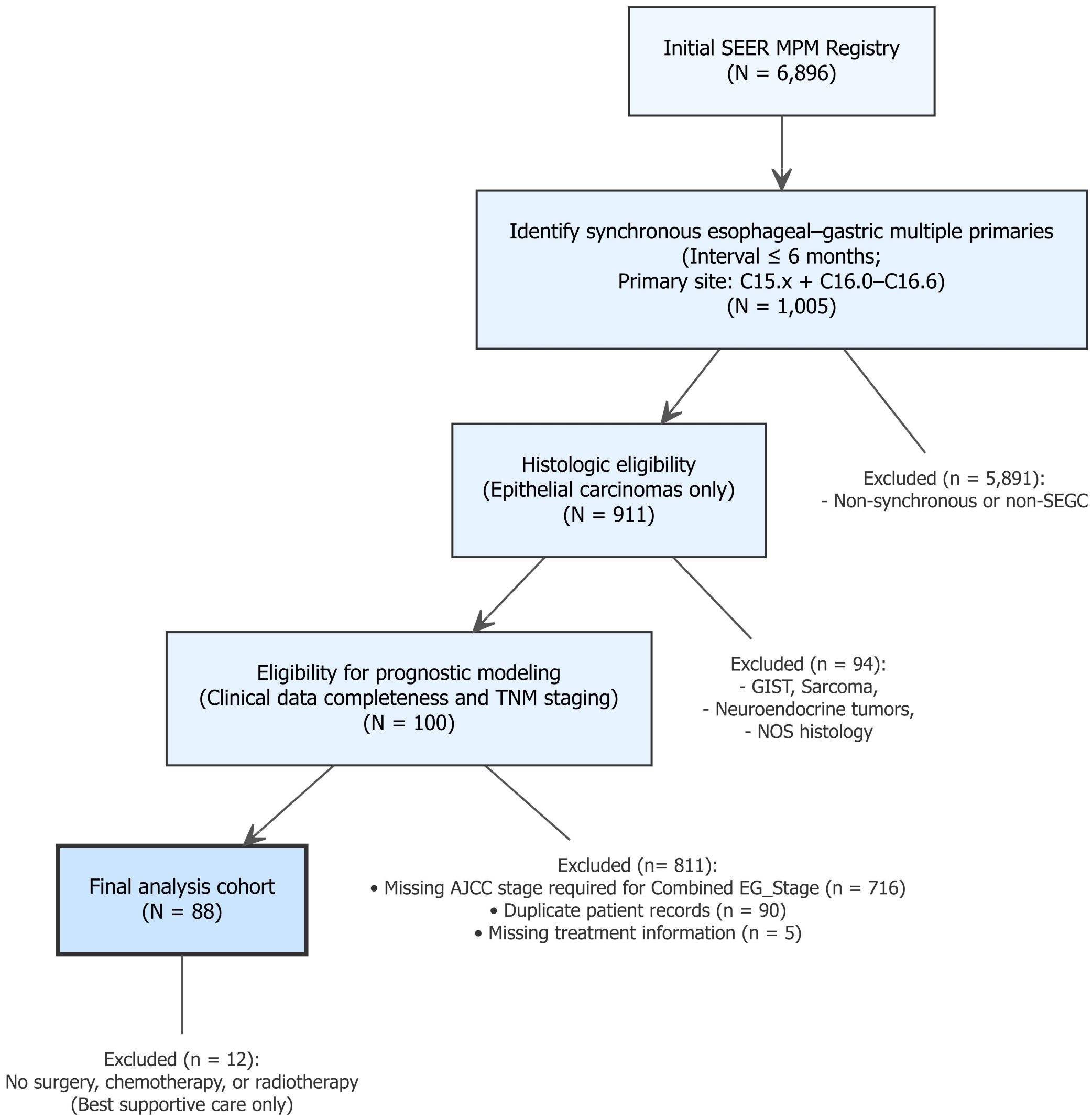

Supplementary Figure 1. Flowchart of the patient selection process for the study cohort. The diagram illustrates the stepwise inclusion and exclusion criteria applied to the initial SEER Multiple Primary Malignancies (MPM) registry. The selection process began with 6,896 patients, identifying those with synchronous esophageal−gastric malignancies (interval less than 6 months; primary site codes C15.x and C16.0 − C16.6). After excluding non-synchronous cases and non-epithelial histologies (e.g., GIST, sarcoma, neuroendocrine tumors), remaining cases were further assessed for availability of essential variables required for model construction, including histologic subtype, AJCC staging, treatment information, and survival outcomes. A complete-case analysis approach was applied, and no imputation of missing data was performed. Finally, patients receiving only best supportive care were excluded to ensure consistency with the actively treated population, yielding a final analysis cohort of 88 patients.

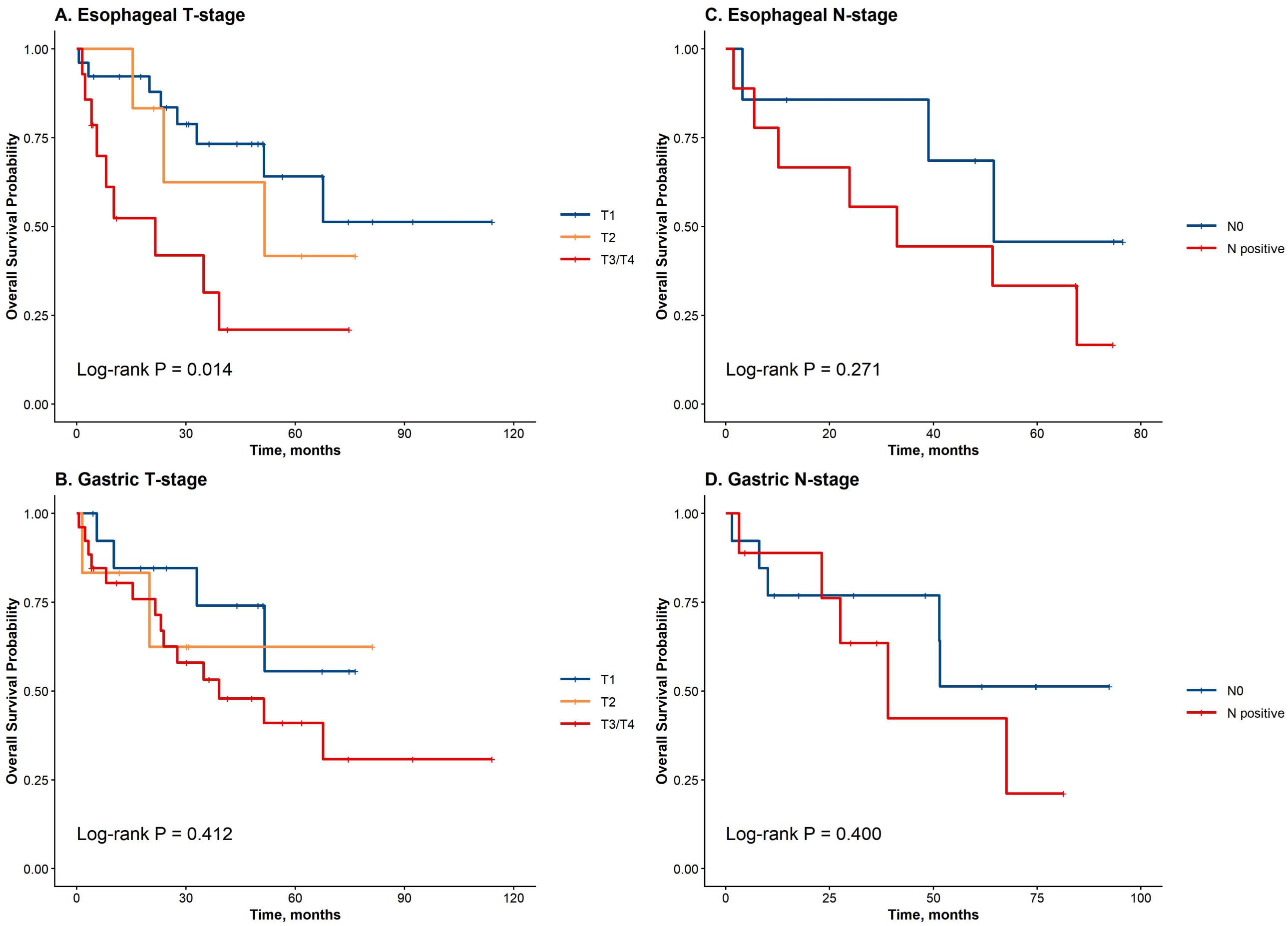

Supplementary Figure 2. Exploratory Kaplan−Meier survival analyses for individual T and N categories in the development cohort. (A) Esophageal T categories (T1 vs. T2 vs. T3–4) demonstrated a statistically significant and monotonic prognostic separation (log-rank P = 0.014), with T1 showing the most favorable survival pattern and T3–4 the poorest outcomes. This pattern is consistent with the observed stratification structure underlying the simplified T-based dichotomy. (B) Gastric T categories showed overlapping survival trajectories without statistically significant differences (log-rank P = 0.412). (C) Esophageal nodal stage (N0 vs. N+) showed a numerically apparent but statistically non-significant separation (log-rank P = 0.271), which may reflect limited statistical power due to small subgroup sizes in this rare disease cohort. (D) Gastric nodal stage similarly showed no statistically significant discrimination (log-rank P = 0.400). Collectively, these exploratory analyses highlight the heterogeneity and inconsistent prognostic performance of granular T and N stratifications, thereby providing contextual justification for the adoption of a simplified dichotomous Combined EG_Stage framework in subsequent analyses.

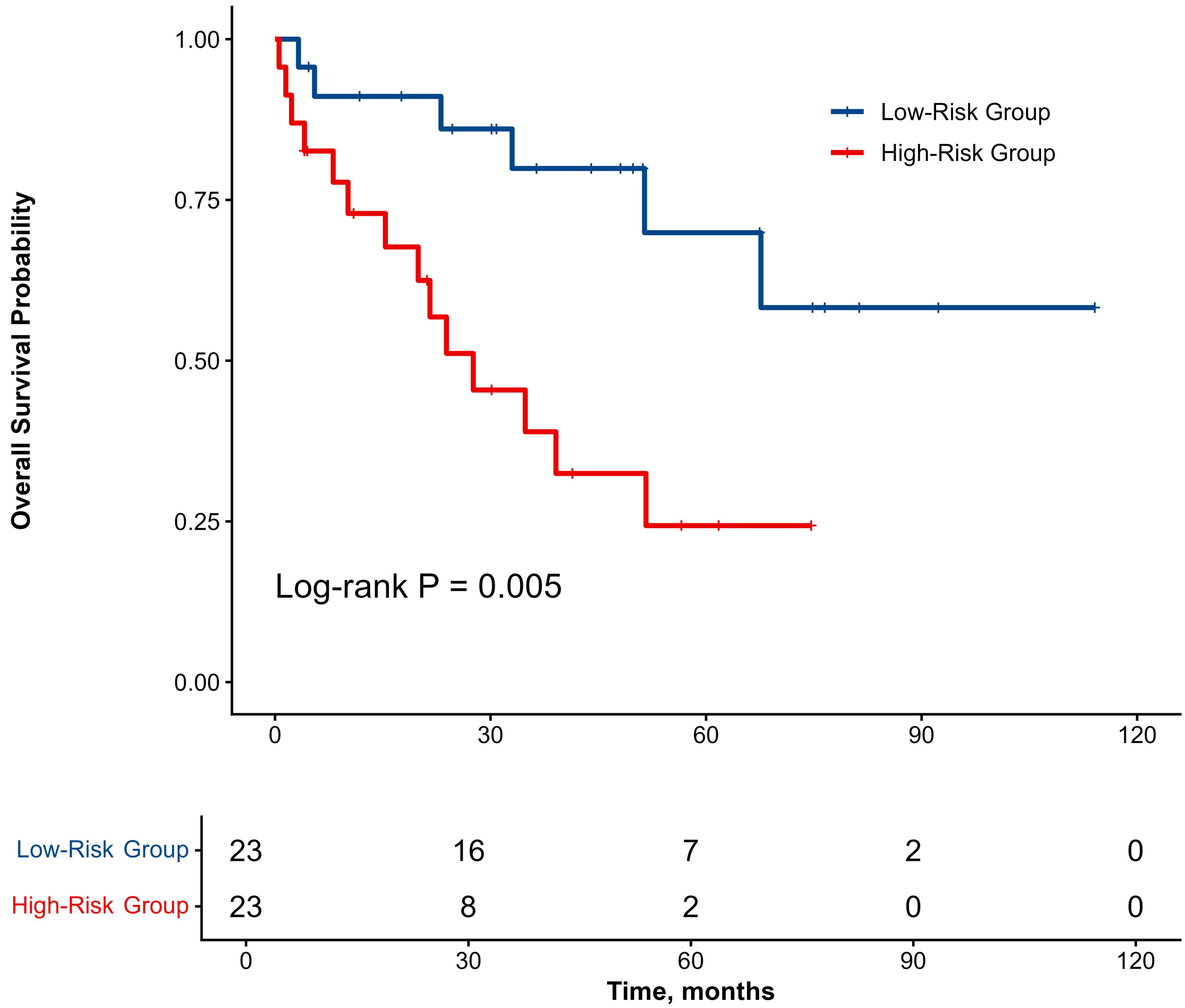

Supplementary Figure 3. Risk stratification and internal discriminative performance of the finalized nomogram. The nomogram demonstrated statistically significant separation between risk strata (log-rank P = 0.005), supporting its internal discriminative performance. Given the relatively low events-per-variable ratio in the development cohort, this apparent stratification should be interpreted with caution, as it may reflect some degree of model optimism and limited stability in small-sample settings. Tick marks indicate censored observations. Numbers below the curves represent patients at risk at each time interval.

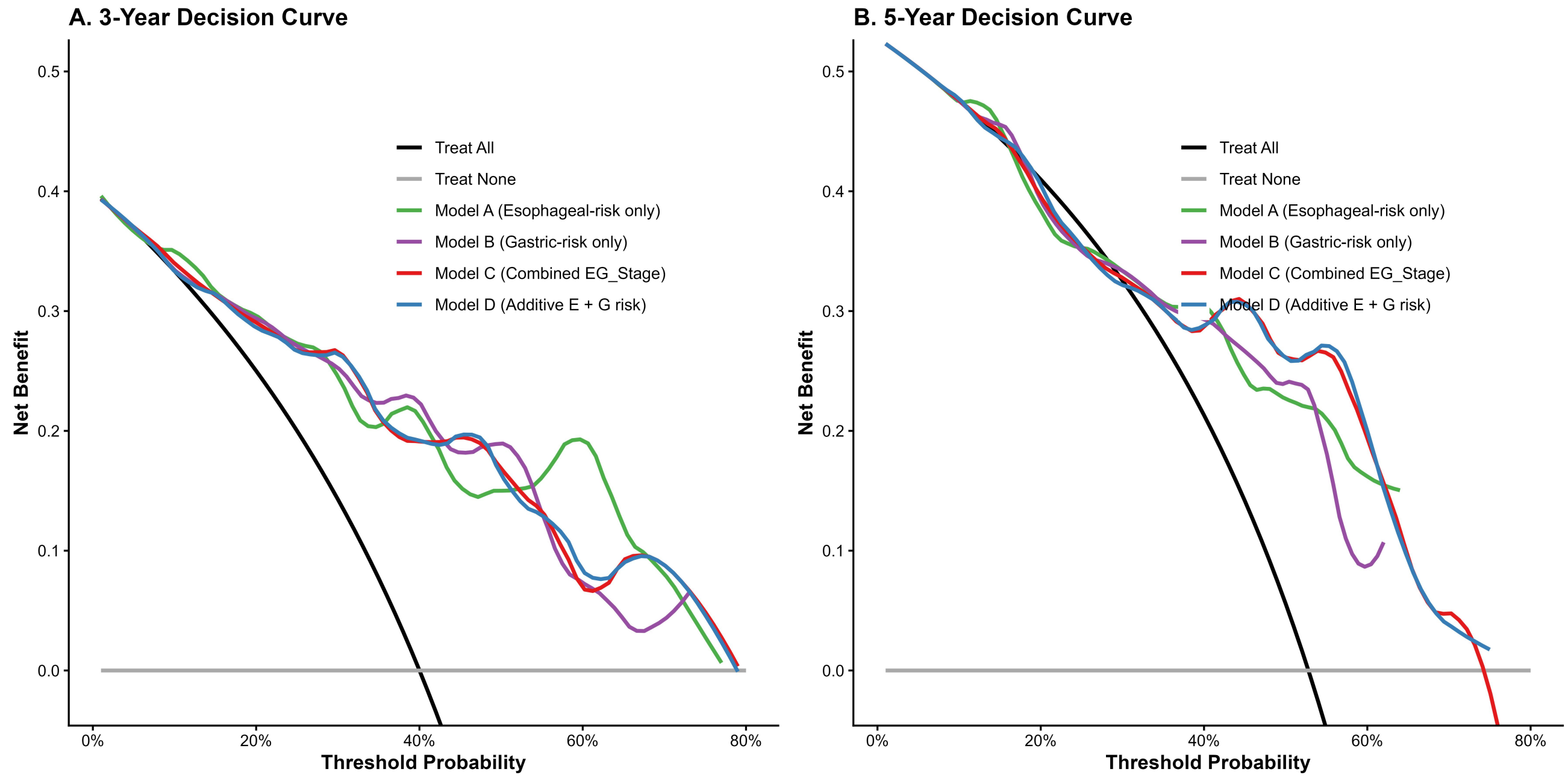

Supplementary Figure 4. Decision curve analysis (DCA) of prognostic models for 3-year (A) and 5-year (B) overall survival horizons. Clinical net benefit was evaluated across a range of threshold probabilities for four prognostic strategies: Model A (Esophageal-risk only), Model B (Gastric-risk only), Model C (Combined EG_Stage), and Model D (additive E-risk + G-risk). The black line represents the "treat-all" strategy, whereas the grey line indicates the "treat-none" strategy. Threshold probabilities between 10% and 70% were considered clinically actionable. Within this range, the net benefit curves demonstrate modest clinical utility for the Combined EG_Stage model, with no clear or consistent superiority identified over alternative staging systems. These findings indicate limited incremental clinical net benefit in active decision-making, suggesting that the primary value of the proposed framework lies in providing a simplified and clinically interpretable tool for exploratory risk stratification in this rare disease setting, rather than serving as a definitive guide for treatment selection.

2 Supplementary Table

The supplementary table has been submitted as a separate file.
